# Effects of CeO_2_ and Sb_2_O_3_ on the Nonlinear Photochemical Process in Ultrashort Laser Gaussian—Bessel Beams Irradiated Photo—Thermo—Refractive Glass

**DOI:** 10.3390/mi12060615

**Published:** 2021-05-26

**Authors:** Xu Wang, Guodong Zhang, Guangying Li, Rui Lou, Zhe Sun, Xiaoping Xie, Weinan Li, Guanghua Cheng

**Affiliations:** 1 State Key Laboratory of Transient Optics and Photonics, Xi’an Institute of Optics and Precision Mechanics of CAS, Xi’an 710119, China; wangxu@opt.cn (X.W.); li_guang_ying@163.com (G.L.); lourui0423@163.com (R.L.); xxp@opt.ac.cn (X.X.); 2 University of Chinese Academy of Sciences, Beijing 100049, China; 3 Electronic Information College, Northwestern Polytechnical University, Xi’an 710072, China; guodongzhang@nwpu.edu.cn; 4 Abbe Center of Photonics, Institute of Optics and Quantum Electronics, Friedrich Schiller University, Max Wien Platz 1, 07743 Jena, Germany; zhe.sun@uni-jena.de

**Keywords:** ultrashort laser, Gaussian—Bessel beam, photo—thermo—refractive glass, photochemical process, microstructure fabrication

## Abstract

Microfluidic chips and optical elements can be fabricated based on the nonlinear photosensitivity in photo–thermo–refractive (PTR) glass by controlling the growth of nanocrystals in the femtosecond (fs) laser–irradiated region. Here, we focus on CeO_2_ and Sb_2_O_3_ that play important roles in UV irradiation, experimentally investigate the effects of the dopants on the nonlinear photochemical process in PTR glass triggered by fs Gaussian–Bessel beams. The results show that the generation of Ag^0^ atoms and the Ag nanoparticles can be improved by CeO_2_ and Sb_2_O_3_ co–doping. Besides, each multivalent ion in PTR glass possibly participates in the electron transfer processes and contributes to the generation of Ag^0^ atoms. Finally, X–ray diffraction analysis reveals the precipitation of NaF nanocrystals with an average size of 10 to 12 nm after laser irradiation and thermal treatment, which is unrelated to the dopants.

## 1. Introduction

Photo–thermo–refractive (PTR) glass is a widely used multifunctional transparent material for recording volume holographic gratings [1,2,3] and fabricating a variety of integrated optical elements [4,5]. The excellent manifold properties of PTR glass are chiefly determined by its unique photosensitiveness [6] that is related to the dopants of Ag_2_O, CeO_2_, Sb_2_O_3_, and SnO_2_ in trace amounts. Among these dopants, Ag_2_O offers Ag^+^ ions that can capture electrons and transform them to neutral Ag^0^ atoms, which prepare for nucleating in the annealing process [7]. CeO_2_ offers Ce^3+^ ions as photosensitizers that can be photoionized by UV irradiation and release photo–electrons [8]. Sb_2_O_3_ and SnO_2_ play important roles in maintaining a balance of the redox ratio between the multivalent ions [9]. A study by Magon et al. [10] confirmed that the electrons that originate from the photosensitivity of Ce^3+^ ions are mostly trapped by Sb^5+^ ions instead of Ag^+^ ions at room temperature. Subsequent thermal treatment leads to releasing the trapped electrons from (Sb^5+^)^–^ ions with a further generation of silver molecular clusters (SMCs) and colloidal particles. Then heating to a higher temperature can result in the precipitation of NaF. Actually, the electron transfer between these multivalent ionic species is more complicated because of the various ionic species in PTR glass. The above explanations all originate from the linear photo–thermal mechanism based on the typical UV irradiation.

In recent years, ultrashort laser photoinscription of glasses has been facilitated a robust development for integrated photonics, which can confine energy to specified three-dimensional geometries well into nanoscale [11,12,13]. This technique supports the modification of electronic properties, structure, and refractive index in glasses based on the nonlinear effects [14]. Therefore, some research about nonlinear photosensitivity in PTR glass with an ultrashort laser were conducted. Glebov et al. [15,16] showed the nonlinear photosensitivity of PTR glass due to the nonlinear ionization of the glass matrix instead of Ce^3+^ ions. They also concluded that multi–photon and tunneling ionization contribute to nonlinear photosensitivity. It provides an alternative method for the fabrication of optical elements, such as phase Fresnel lens [17], transmission volume phase holographic gratings [18], and waveguides [19], in PTR glass. In our previous work [20], the nonlinear interactions between PTR glass and ultrashort laser Gaussian–Bessel (GB) beams [21,22] were studied. The results showed the evolution of nanocrystals in the irradiated area and the regulation of Ag nanoparticles (NPs) through laser parameters. The mechanism of the growth of nanocrystals and the refractive index modifications in three–dimension is a prerequisite for achieving precise control of the size and concentration of the nanocrystals, expanding the application of PTR glass in the field of integrated optical devices. The diffraction properties of volume Bragg gratings in PTR glass can be optimized by controlling the crystallization characteristics. Moreover, the growth (size and concentration) of nanocrystals in PTR glass could affect the performance of microfluidic devices. Additionally, the research into the refractive index change is valuable for designing the optical waveguides. However, the origin and migration of the electrons are complex in the ultrashort laser irradiation process. Whether the Ce^3+^ and Sb^5+^ ions take part in the nonlinear photo-thermal process and influence the precipitation of Ag NPs even the growth of NaF is still unclear.

In this work, PTR samples with different doping have been prepared. We investigated the nonlinear photochemical processes in different samples based on fs laser GB beams irradiation. The refractive index modification and the absorption spectra are compared and analyzed before and after thermal treatment. The effects of CeO_2_ and Sb_2_O_3_ on the formation and evolution of defects and SMCs are analyzed by electron paramagnetic resonance (EPR) spectra. Furthermore, the crystallization behaviors are studied and confirm that fs laser can trigger the formation of the core–shell nanocrystals whether CeO_2_ or Sb_2_O_3_ is doped.

## 2. Experimental

### 2.1. Materials

A group of PTR glass samples based on the 73SiO_2_–11Na_2_O–7(ZnO+Al_2_O_3_)–3(BaO+La_2_O_3_)–5NaF–1KBr (mol%) matrix (labeled as P), with different doped small additions of SnO_2_, CeO_2_, Sb_2_O_3_, and AgNO_3_ were synthesized and used in this work. The codes and corresponding compositions of the dopants are presented in Table 1. First, the mixed raw materials were mixed and melted in a platinum crucible at 1440 °C for 5 h. Subsequently, the molten glass was cast into a copper mold and then annealed at 500 °C (near the glass transition temperature, T_g_, shown in Table 1) for 6 h. Finally, all the samples used in this work were cut with a size of 4 mm × 4 mm × 2 mm and precisely polished using CeO_2_ microparticles.

### 2.2. Fabrication of Microstructures with GB Beams

**Figure 1:**
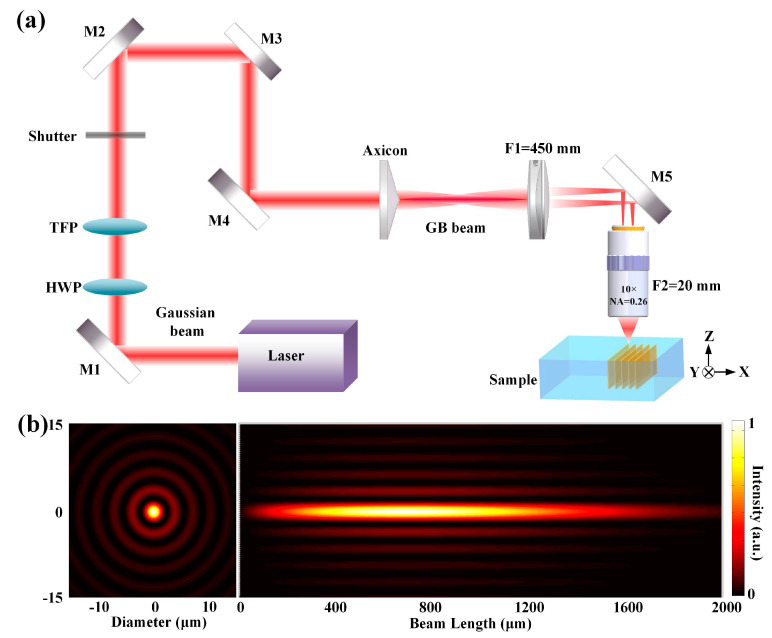
(**a**) Schematic of the setup for inducing microstructures on PTR glass, the GB beam generated using an axicon (nominal base angle = 1°). (**b**) Simulations of the transverse and axial intensity distribution of the GB beam in the PTR glass under 220 fs and 4 μJ.

An amplified Yb: KGW femtosecond laser oscillator (Pharos, Light Conversion, Lithuania) was employed as the irradiation source with 220 fs, 100 kHz, and 1030 nm. The schematic of the fs–laser fabrication is shown in Figure 1a. The pulse energy can be continuously adjusted utilizing a thin–film polarizer (TFP) and a half–wave plate (HWP). An axicon with a nominal base angle = 1° was used for providing a first Bessel region. Further, a 4f system (a factor of 22.5) composed of a convex lens (f1 = 450 mm) and a microscope objective (10×, NA = 0.26, f2 = 20 mm, Minutoyo NIR, Japan) was employed to get a second Bessel region adapted for micromachining [23]. Figure 1b presents the simulations of the transverse and axial intensity distribution of the GB beam in the PTR glass. The initial Gaussian beams had a radius of 3.8 mm, while the central lobe of the GB beam was about 4.4 μm in diameter at full width at half maximum (FWHM). The length of filament in PTR glass was around 2 mm from a side view. The PTR glass was fixed on a high–precision motorized 3D mobile platform (ANT130, Aerotech, Pittsburgh, PA, USA). By translating the samples perpendicularly to the GB beam at a speed of 400 μm/s, a range of parallel filaments with interline distances of 5 μm and length of 4 mm were written 150 μm below the surface, resulting in square patterns. The pulse energy was fixed at 4 μJ/pulse. After laser exposure, the samples went through thermal treatment at 460 °C for 5 h and 540 °C for 3 h in a muffle furnace.

### 2.3. Characterization Techniques

The morphological characteristics of structures were observed using a positive phase contrast microscope (PCM, BX51, Olympus, Tokyo, Japan) to access the refractive index changes. The optical transmission measurements were performed with a UV–VIS–NIR spectrophotometer (UV–3101, Shimadzu, Japan) in the range of 200 to 800 nm. EPR spectra were performed at 100 K using a helium–flow cryostat with an EPR spectrometer (ELEXSYS E500, Bruker, Germany), operating at the X–band (9.44 GHz) with 100 kHz field modulation. The phase composition of the samples was analyzed using an X–ray diffractometer (XRD, D8 Discover, Bruker, Germany) with CoKα radiation in the 2θ range from 40° to 90° with an increment of 0.05°.

## 3. Results and Discussion

### 3.1. Refractive Index Changes in PTR Glass Samples

**Figure 2:**
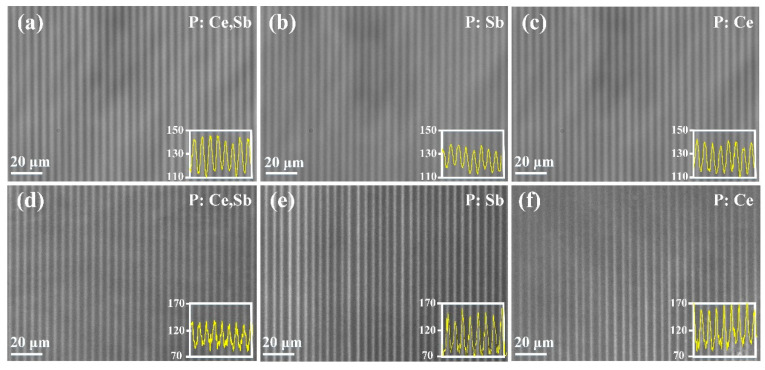
The PCM images of the microstructures in XY–plane: (**a**–**c**) after irradiation; (**d**–**f**) after irradiation and thermal treatment. The insets show the corresponding gray level distribution curves of the framed areas.

To study the difference of induced refractive index changes in the doped and undoped samples, we performed the PCM images of the microstructures in XY–plane after fs–laser exposure and subsequent thermal treatment process shown in Figure 2. In this arrangement, the black zones represent relative positive index modifications, and white regions indicate negative changes. It can be found that fs–laser irradiation led to the darkened tracks in PCM of all the samples, as shown in Figure 2a–c. The widths of the exposed regions were the same for each case (about 1 μm). Further, the darkened tracks implied type I photoinscription and indicated a soft photochemically induced refractive index increment. The increase can be induced by the structural densification process mainly caused by defects [24,25]. Meanwhile, this phenomenon implied the refractive index change of PTR glass during the fs–laser exposure process was non–negligible (Δn, 10^−4^~10^−3^) [13,26], which was totally different from the change in the case of UV exposure (Δn, 10^−6^) [27]. The insets (yellow curves) are the gray level distribution curves of the framed areas. As for P: Ce, Sb, and P: Ce samples, the traces showed a rather higher contrast compared to P: Sb. The increase in the contrast is probably associated with the larger quantity of the defects induced by laser irradiation when doping with Ce^3+^ ions due to the increased probability of nonlinear ionization, and the introduction of Sb_2_O_3_ showed a slight enhancement effect. After thermal treatment, the darkened traces totally change to white, indicating the decrement of the refractive index with respect to the matrix, as shown in Figure 2d–f. We proved such treatment of P: Ce, Sb sample can lead to the generation of NaF (*n_NaF_*~1.33) nanocrystals that had a lower refractive index compared to the matrix (*n_PTR_*~1.49) [28]. Therefore, the nanocrystals and the high residual stresses [29] around them are considered the major contributors to the decrease. Based on this mechanism, we have reason to speculate that the reduction of refractive index in P: Sb and P: Ce samples probably results from the same cause. Moreover, P: Sb and P: Ce showed a relatively higher negative refractive index contrast and nonuniformity of the structure than P: Ce, Sb, which can be associated with the density of the nanocrystals and will be our future work.

### 3.2. The Transmission and Absorption Spectra Analysis

**Figure 3:**
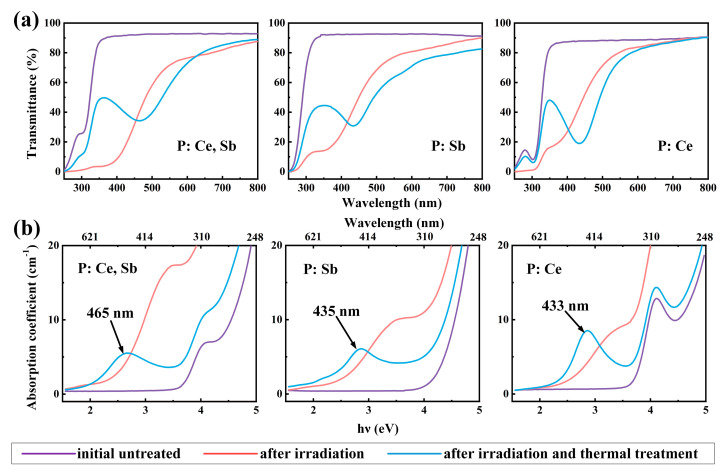
(**a**) Transmission spectra and (**b**) absorption spectra of P: Ce, Sb, P: Sb, and P: Ce samples.

In order to reduce the loss of the grating pattern during the transmission spectrum measurement, we placed the samples near the detector input port in the sample cell and customized accessories, including a sample holder and a diaphragm, to ensure the effective collection of the first- and second-order diffraction light. The transmission curves of the pristine glass samples, the fs laser exposed samples, and the fs laser exposed with heat-treated samples are shown in Figure 3a. The calculated absorption curves are presented in Figure 3b. It can be found that all the initial untreated samples kept a high transmittance in the range from 350 to 800 nm. The bands located at around λ = 302 nm (E = 4.11 eV) of CeO_2_ doped samples were attributed to Ce^3+^ ions arising from the 4f–5d transitions. Sb_2_O_3_ can improve the transmittance as a clarifying agent. Fs–laser radiation resulted in similar decline trends for all samples. Based on the decomposition of absorption spectra with Gaussian functions established in our previous work [20], the bands at 392 nm and 441 nm were attributed to the SMCs because of the successive pulses’ cumulative thermal effects on the focal volume. At the same time, the band around 350 nm could be attributed to color centers due to the lattice defects or impurities in PTR glass that can capture the free electrons and holes. Therefore, the obvious decrease in the transmittance in the UV and visible range indicates the generation of color centers and SMCs [30]. After heat treatment, a noticeable reduction in the transmittance in the 350 nm–600 nm range, the absorption at 450 nm was caused by the formation of Ag NPs [31] due to their surface plasmon resonance [31].

**Figure 4:**
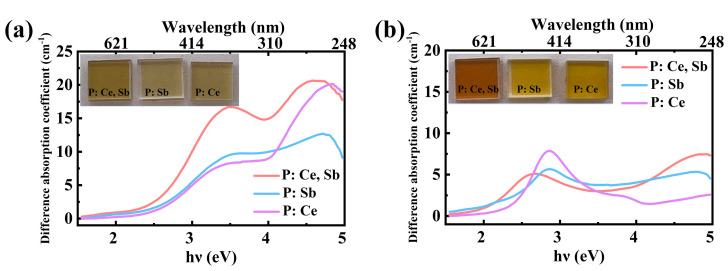
The difference absorption spectra of the glass samples: (**a**) after irradiation; (**b**) after irradiation and thermal treatment. The insets show the photographs of the corresponding glass samples.

To investigate the absorption spectral changes in more detail, we also present the absorbance after deducting the absorbance of the initial untreated samples, as shown in Figure 4. It can be found that laser irradiation resulted in absorption increases at around λ = 260 nm (E = 4.78 eV) by doping with CeO_2_, and another λ = 353 nm (E = 3.52 eV) by co-doping with Sb_2_O_3_ in CeO_2_ doped sample. These results indicate that the formation of color centers as well as the SMCs can be significantly prompted by doping both (P: Ce, Sb). Furthermore, a portion of the absorption could also be attributed to Ce^4+^ and Ce^3+^ ions, which implies the ions probably take part in the nonlinear photosensitive process. This phenomenon was in accordance with the change of color of the samples in Figure 4a (inset). Compared with P: Sb and P: Ce, the yellow coloration of P: Ce, Sb had a darker appearance, indicating higher efficiency of transformations of charged Ag^+^ ions to Ag^0^ atoms and SMCs (Ag_2_, Ag_2_^+^, Ag_3_^+^, etc.). Subsequent thermal treatment led to darker colors of the samples (Figure 4b), particularly for P: Ce, Sb sample, reddish–brown emerges. The absorption bands at around λ = 465 nm (E = 2.67 eV), λ = 435 nm (E = 2.86 eV), and λ = 433 nm (E = 2.87 eV) were the SPR bands of Ag NPs, which were primarily dependent on the size distribution, the concentration, and the shape [11]. The mean diameters of Ag NPs are about 2–3 nm based on Mie–Drude theory [32]. And according to the area of the absorption peak of Ag NPs, the concentration of Ag NPs decreased significantly for P: Sb.

### 3.3. EPR and XRD Analysis

**Figure 5:**
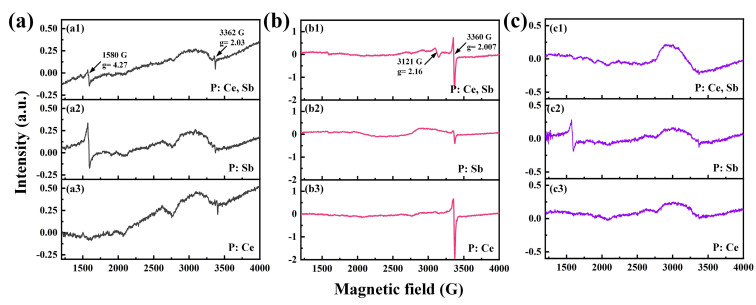
Measured EPR spectra of glass samples: (**a**) initial untreated; (**b**) after irradiation; (**c**) after irradiation and thermal treatment.

To further obtain specific structural information about the role of these dopants during the nonlinear photo–thermal process, samples were ground into a powder with the same mass and placed in quartz tubes. Continuous–wave EPR measurements of the powdered samples are displayed in Figure 5. A clear, sharp signal was observed in the pristine P: Ce, Sb sample (Figure 5(a1)), in which the magnetic field was around 1580 G, corresponding to the electron’s g–factor of around 4.27. Meanwhile, there was also a weak signal around 3362 G (g = 2.03), and both signals can be attributed to Fe^3+^ ions [33] that were introduced in the refining process. Significant changes occurred in the EPR spectra of the fs laser–irradiated P: Ce, Sb sample in comparison with the pristine sample (Figure 5(b1)). A new signal near 3121 G (g = 2.16) appeared that can be associated with the superimposing signals of Ag^0^ neutral atoms and Ag_2_^+^ molecular ions [34]. In addition, another new sharp signal near 3360 G (g = 2.007) can be assigned to Si–E′ centers [35]. Other defects that existed in our glass sample could not be detected here because of the experimental limitations. Then further thermal treatment (Figure 5(c1)) resulted in the near disappearance of the defect signal. Similarly, the pristine P: Sb sample (Figure 5(a2)) owned a relatively intense signal of Fe^3+^ ions, while a weak signal of Si–E′ for the irradiated sample Figure 5(b2)). The signal of Fe^3+^ ions still existed obviously after thermal treatment (Figure 5(c2)). As for the P: Ce sample, the signal of Fe^3+^ ions was weak before irradiation (Figure 5(a3)), while the signal of defects was apparent after irradiation (Figure 5(b3)). Almost no signals could be detected after thermal treatment (Figure 5(c3)).

Comparing the signals between different samples in different states, we found that the signals of Fe^3+^ ions fluctuated randomly for the initial untreated glass samples due to the non-controlled impurity ions. This contamination existed in these glasses at a very low level but still played a role in an electron acceptor under UV irradiation [9]. After fs–laser irradiation, the defect signals of P: Ce and P: Ce, Sb samples were much stronger than the P: Sb sample, which suggests that the addition of CeO_2_ can increase the fs–laser radiation-induced defects in the PTR glass. The signal of Ag^0^ neutral atoms and Ag_2_^+^ molecular ions could only be detected in the P: Ce, Sb sample. This phenomenon can be explained by the increase in the number of transformations of Ag^+^ ions into Ag^0^ atoms. Ag^+^ ions can capture the electrons excited by fs–laser irradiation directly, as well as Ce^4+^, Sb^5+^, and Sn^4+^ ions. As for electron transfer processes in UV–irradiated PTR, electrons are transferred to Ag^+^ ions and only occur in the subsequent heat treatment when the temperature increases to a few hundred degrees [36]. In our case, ultrashort laser pulses could produce a high temperature during the irradiation, which could transfer electrons from [Ce^4+^]e^−^, (Sb^5+^)^−^, and (Sn^4+^)^−^ ions to more Ag^+^ ions. These results agreed well with the absorption spectra in Figure 4a. Further thermal treatment resulted in the disappearance of the defect signals due to the bleaching of the defects by employing high–temperature annealing. EPR spectra also suggested that Fe^3+^ ions could take part in the electron transfer process because of the slight change in the signal of Fe^3+^ ions. The mechanism has not been studied in–depth and will be the next step of the work. Hence, the origin of electrons and their transfer process could be related to these different multivalent ions in PTR glass undergoing fs–laser irradiation.

**Figure 6:**
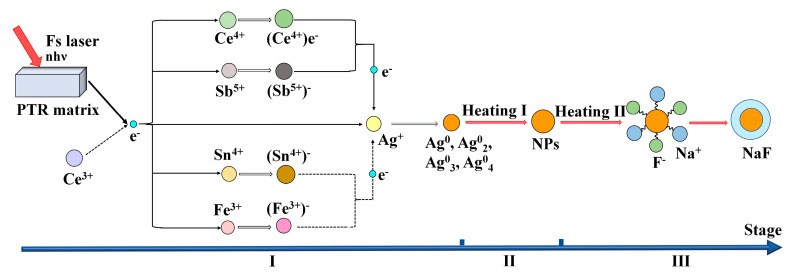
Schematic of the nonlinear photo–thermo–induced crystallization mechanism in P: Ce, Sb sample.

We propose the nonlinear photo–thermo–induced crystallization mechanism based on fs–laser irradiation in the P: Ce, Sb sample, as shown in Figure 6. In the first stage, fs-laser irradiation can trigger the nonlinear effects in the PTR glass matrix, a quantity of free electron-hole pairs can be produced. Ce^3+^ ions also have the opportunity to absorb photon energies to release electrons. All the photoinduced electrons can be captured by Ag^+^, Ce^4+^, Sb^5+^, Sn^4+^, and Fe^3+^ ions. Then [Ce^4+^]e^−^, (Sb^5+^)^−^, (Sn^4+^)^−^, and (Fe^3+^)^−^ ions could transfer electrons to Ag^+^ ions. CeO_2_ and Sb_2_O_3_ dominate the electron transfer process according to the absorption and EPR spectra. The second and third stages (Heating I and Heating II) are responsible for repairing part of color centers, further growth of Ag NPs, and the formation of the NaF nanocrystals.

**Figure 7:**
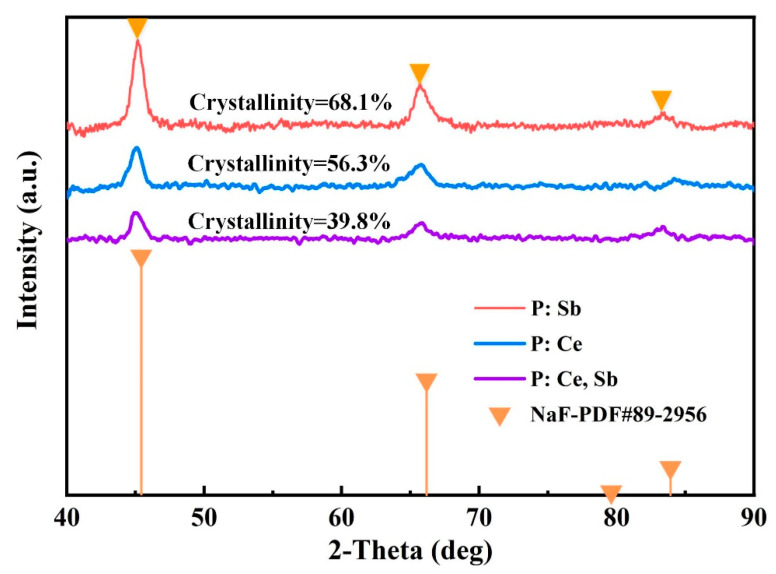
XRD patterns of the PTR glass samples after fs–laser exposure and thermal treatment.

The powdered samples were also compared according to the XRD analysis shown in Figure 7. The original glass samples presented their amorphous nature. Nevertheless, the modified samples with laser exposure and heat treatment showed apparent crystal structure. The main diffraction peaks of NaF were observed at 45.15°, 66.11°, and 83.75°, which match the (200), (220), and (222) planes of NaF (ICSD card files nos. 89–2956). This identifies that such treatment of samples leads to the formation of NaF whether or not CeO_2_ or Sb_2_O_3_ is doped. Owing to the slight difference in full width at half maximum (FWHM), the particle size was estimated to be about 10–12 nm from the diffraction peaks using Scherrer’s equation [37] for all samples. However, the intensity of crystalline peaks changed obviously and decreased by a large margin for P: Ce, Sb. The degree of crystallinity [38] can be calculated from the area of crystalline peaks and divide by the area of all peaks (including crystalline and amorphous). The values commented on the corresponding curves showed that the P: Sb sample possessed higher crystallinity, which suggests that the degree of crystallinity could not be improved by doping CeO_2_, even the P: Ce, Sb sample could generate more SMCs during the irradiation process. Prolonging the treatment time may enhance the crystallinity in a certain range [39], as more SMCs may be fit for the thermal treatment with an extended period. The detailed kinetics of NaF crystallization in fs exposed PTR glass is complicated and needs more theory and experiment to research, which will be our future work.

## 4. Conclusions

In summary, we experimentally investigated the effects of CeO_2_ and Sb_2_O_3_ in PTR glass matrix on the nonlinear photochemical process under fs GB beams irradiation. The addition or deleting of CeO_2_ and Sb_2_O_3_ in PTR glass led to non–negligible changes in the formation and evolution of silver–containing nanocrystals, which were manifested in the refractive index, transmission and absorption spectra, EPR spectra, and XRD. The characterization proved that the addition of CeO_2_ and Sb_2_O_3_ could induce a relatively higher refractive index increment and offer more SMCs, even Ag NPs. In addition, nanocrystals with a shell of NaF and an average size of 10 to 12 nm can be obtained whether CeO_2_ or Sb_2_O_3_ is doped. Meanwhile, the high concentration of SMCs is not a prerequisite for the high degree of crystallinity by XRD analysis. An in–depth understanding of the nonlinear photosensitivity in PTR glass with ultrashort laser GB beams, including the growth of nanocrystals and the controllable refractive index modifications in three–dimension, which are meaningful for designing and fabricating microstructures, has been presented. Therefore, the above results may be used in integrated optical devices, such as high–efficiency volume Bragg gratings, microfluidic chips, and optical waveguides.

## Figures and Tables

**Table 1:** The compositions of the dopants in PTR glass samples.

Code	Dopant Contents, mol%				T_g_, °C
	SnO_2_	AgNO_3_	CeO_2_	Sb_2_O_3_	
P: Ce, Sb	0.02	0.01	0.02	0.08	509.7
P: Sb	0.02	0.01	–	0.08	488.6
P: Ce	0.02	0.01	0.02	–	504.2
